# Contemporary crustal movement of southeastern Tibet: Constraints from dense GPS measurements

**DOI:** 10.1038/srep45348

**Published:** 2017-03-28

**Authors:** Yuanjin Pan, Wen-Bin Shen

**Affiliations:** 1 School of Geodesy and Geomatics, Wuhan University, Wuhan, 430079, China; 2 State Key Laboratory of Information Engineering in Surveying, Mapping and Remote Sensing, Wuhan University, Wuhan, 430079, China.

## Abstract

The ongoing collision between the Indian plate and the Eurasian plate brings up N-S crustal shortening and thickening of the Tibet Plateau, but its dynamic mechanisms remain controversial yet. As one of the most tectonically active regions of the world, South-Eastern Tibet (SET) has been greatly paid attention to by many geoscientists. Here we present the latest three-dimensional GPS velocity field to constrain the present-day tectonic process of SET, which may highlight the complex vertical crustal deformation. Improved data processing strategies are adopted to enhance the strain patterns throughout SET. The crustal uplifting and subsidence are dominated by regional deep tectonic dynamic processes. Results show that the Gongga Shan is uplifting with 1–1.5 mm/yr. Nevertheless, an anomalous crustal uplifting of ~8.7 mm/yr and negative horizontal dilation rates of 40–50 nstrain/yr throughout the Longmenshan structure reveal that this structure is caused by the intracontinental subduction of the Yangtze Craton. The Xianshuihe-Xiaojiang fault is a major active sinistral strike-slip fault which strikes essentially and consistently with the maximum shear strain rates. These observations suggest that the upper crustal deformation is closely related with the regulation and coupling of deep material.

India began colliding with Eurasia over 40–50 million years ago, leading to strong North-South crustal shortening and uplifting^1^,^2^. In addition, the generation of blocks and strike-slip faults is mainly due to that material migration of surface and deep lithosphere throughout Tibet. The east-west trending crustal extension has dominated the tectonic processes of the sinistral strike-slipping^3^. The southeast boundary of Tibet is one of the areas, where earthquake activity is strongest with greatest geological hazards in the intra continent of China, and it is related to the isostatic adjustment of the interior material movements of the whole Tibet^4^,^5^. The current models to explain the tectonic processes and mechanical evolution of the plateau are attributed to either a rigid block-slip extrusion along boundary faults in the upper crust^6^,^7^ or a narrowly distributed viscous channel flow in the lower crust^3^,^8^. Therefore, the South-Eastern Tibet (SET) is an important passageway for materials moving toward southeast and also with exerting profound influences on tectonic evolution and regional crustal deformation.

The southeast borderland of Tibet is a seismically active zone and exhibits complex topographic relief, as shown in Fig. 1(a). Two big earthquakes occurred in this region during 1976–2016, i.e. the 2008 Wenchuan and 2011 Burma/China earthquakes which reveal evidences for active thrust and strike-slip faulting. Seismic data provide key information on the crustal structure at depth^9^, and suggest that the Yangtze sub-continent crust extends beneath this region^10^. The magnetotelluric data also suggest that there are two major zones or channels with material flowing in orogenic belts, contributing to the uplift of the plateau^4^. The receiver functions and surface wave dispersion data clearly imaged the middle-to-lower crust in SET, suggesting two flow channels of the middle-to-lower crustal materials extruding from north toward southeast and southwest of Tibet^9^,^11^,^12^. Crustal motion is also characterized by extensive strike-slip faults and shear zones along major tectonic boundaries at the surface^2^,^13^,^14^. These rapid tectonic movements could be related to the current orogenesis caused by the Indian-Eurasian collision, and by contrast, flow in the asthenosphere may be related to the absolute motion of Eurasia^15^.

Global Position System (GPS) measurements have been widely used to constrain the horizontal crustal deformation of Tibet^16^,^17^,^18^,^19^, indicating that Tibet is currently undergoing eastward block motion and clockwise rotation due to the ongoing collision between the Indian and Eurasian plate. Meanwhile, the vertical crustal deformation throughout Tibet at different spatial and temporal scales (e.g. surface short-term and long-term crustal deformation) brings us new insights on the dynamics of contemporary tectonic processes^20^,^21^,^22^,^23^,^24^,^25^. However, how the crustal three-dimensional (3D) deformation varies and the dynamics mechanism of underlying material changes in and around Tibet remains controversy^8^,^14^,^18^.

In this study, we integrated GPS data with an improved data processing strategy to assess the 3D velocity throughout SET. The GPS-derived horizontal velocity field relative to the stable Eurasian plate displays crustal block motion by clockwise rotation around the Eastern Himalayan Syntaxis. Particularly the regional vertical-improved GPS velocity including the Longmen Shan zone, Gongga Shan and Southern Yunnan sub-block shows regional crustal vertical deformation properties of SET. Then, the strain rates are calculated from the GPS-derived horizontal velocity. Finally additional information on the methods is described at the end of the paper.

## Results and Discussion

The newly GPS horizontal velocity field relative to the stable Eurasia throughout SET and neighboring regions reveals a continuous crustal clockwise motion around the Eastern Himalayan Syntaxis, as shown in Fig. 2(a). The velocity pattern reflects a lateral extrusion and continuous horizontal deformation of the SET upper crust. Along some eastern boundary belts, Qujiang, Xiaojiang fault belts in the south of Yunnan to Xianshuihe fault belts in northwestern Sichuan, the present horizontal deformation rates decrease from 15–20 mm/yr along the western segment to 5–10 mm/yr along the eastern segment. The slow crustal movements on the Yangtze Craton block as a resistance play an important role in the deformation of SET. The GPS-derived horizontal velocity field displays N-E crustal motions with rates of 10–15 mm/yr, which reveals that crustal extension is dominated in the Songpan-Ganzi Block.

The vertical velocity field is obtained from continuous GPS (CGPS) stations (Fig. 2(b)). The campaign-mode GPS sites were not used here because they might be largely influenced by various geophysical processes (including annual, inter-annual variations and loading effects caused by the intense precipitation, melting glaciers, and rising lakes). The surface loading effects and common mode errors are removed from the time series. The vertical change rates are small along the Xianshuihe and Anninghe fault belts, but the vertical change rates along the Xiaojiang, Longmenshan fault belts and Zemuhe fault (ZMH f.) belts are comparatively large. The Songpan-Ganzi Block (S-G Block) appears to be gradually uplifting at a rate of 1–2 mm/yr and there is week uplifting at a rate of 0–0.5 mm/yr along the Kunlun fault. However, the vertical crustal deformation shows anomalous tectonic uplifting with a rate of 8.7 mm/yr on the Longmen Shan structure due to the ongoing rise of the Longmen Shan mountain range that marks the eastern border of Tibet. The structure of the induced seismicity is located in the central fracture belt along the Longmen Shan structural belt, and the action of the squeeze stress leads to thrust movement from southwest to northeast. The Gongga Shan in the Kangding region, of the southwestern Sichuan basin shows crust uplifting with rates of 1–1.5 mm/yr.

The western corner of SET where the Indian lithosphere subducts beneath Tibet and Burma^27^, shows a significant crustal uplifting with a rate of ~3 mm/yr. Instead, the Lanping-Simao Fold System (LSFS), with respect to the south foot of the Southern Yunnan sub-block (SYB), shows an obvious subsidence with rates between −0.1 and −3 mm/yr (Fig. 2(b)). It suggests that crustal extension leads to lithospheric thinning of the upper crust on the Southwestern Yunnan segment.

In order to identify how much a material has been deformed and where it has been deformed, the strain modeling is facilitated throughout SET. Strain rates are sensitive to the length scale of the analysis. To calculate the average strain rates, two methods are adopted, the Grid-Distance Weighted approach and the Grid-Nearest Neighbor approach^28^. Figures 3(a) and 4(a) do not show local strain anomalies because of the regional smoothing applied. Positive dilatation rates show crustal thinning while negative dilatation rates show thickening (background color in Fig. 3a and b), respectively. The principal strain rates show crustal shortening and extension infinitesimal axes^16^. The dilatational strain pattern suggests that crustal ESE-WNW extension dominates the active deformation of the SET interior, with approximately half accommodated by strike-slip faulting.

The continuous positive extension strain pattern (Fig. 3(a)) reveals continuous crustal extension of the Jiasha River fault around the eastern end of the Himalaya, with rates between 15 to 20 nstrain/yr. The Chuan-Dian Block shows a crustal extension with dilatational rates of 10–15 nstrain/yr. However, the plates underneath between the South China block and eastern Tibet generate a horizontal shortening throughout the Longmen Shan faults. With the Grid-Nearest Neighbor approach analysis, local or regional strain patterns are emphasized. In Fig. 3(b), the strongest negative strain dilatation anomalies are found in the Longmen Shan Fault zone that is related to the Yangtze Craton block subducting under Eastern Tibet. The compressional stress mainly comes from the NW, and acts as a resistance in the SE direction.

The crust SSE- SSW extensional strain dominates active deformation of SET (Fig. 4a and b)). The Sanchakou area, where the Xianshuihe, Longmenshan and Anninghe fault zones strech and converge, is located in a special tectonic region whereas the interior strike-slip faulting dominates. Our maximum shear strain rates imply that the western and eastern boundaries of the Sichuan Yunnan rhombus block, i.e. the Honghe and Xiaojiang faults, might be erection seams or collision belts. Yet the energy source of the Longmen Shan’s southeast pushing might come from the strike of the Indian Plate onto the Eurasian Plate and its northward pushing. The maximum shear pattern maintains consistency with the strike-slip faults, along the Chuan-Dian (C-D) Block with block-wise motion channel, and further indicating that shallow crustal deformation is closely related with the regulation and coupling of deep material, from upper to middle-lower crust.

It is meaningful to study the tectonic deformation of SET in order to understand the dynamic process of the Indian-Eurasian collision, plateau’s uplift and its mechanism. The NNE-SSW shortening of the plateau interior is accommodated by conjugate strike-slip faulting and orthogonal normal faulting^17^. The mountain building occurs in the process of the subduction of the Yangtze block under Eastern Tibet^10^. The lower crustal thickening on the west-side of the Longmen Shan faults is closely related to the surface rapid uplifting^4^. Seismic anisotropy provides key information on the structure and deformation of the deep lithosphere throughout SET, and presents two flow channels of the middle-to-lower crustal materials extruded from Tibet^8^,^12^,^29^,^30^. The GPS-derived horizontal crustal motion revealed a glacier-like flow zone which could be attributed to an eastward escape of highly plastic upper crustal material driven by a lower crust viscous channel flow^3^,^18^,^23^.

## Conclusion

The present-day crustal motion on the basis of the measurements of 409 GPS stations highlights the lithosphere deformation throughout SET and its surroundings. The improved vertical crustal deformation is constrained by removing the surface loadings from CGPS time series, showing a strong correlation with average principal horizontal shortening strain rates. The regional uplifting and sinking of the crust are sensitive to the crustal compression and extension. However, a regional crust pulling-apart induces a sinking of a basin, resulting in multi-periodic activities of its boundary faults and relative uplifting of the mountain, i.e. the Sichuan basin (Fig. 2(b)). In contrast to the GPS-derived unusual crustal vertical deformation this region is affected by the subduction of the Yangtze Craton block beneath the Eurasian plate^10^.

The strike-slip faults play a key role in reconciling the block motions and crustal uplifting of Tibet. The strain shear rates reveal the crustal strike-slipping and extension on the Chuan-Dian Block which is bounded by the left-lateral Xianshuihe-Anninghe-Xiaojiang Faults. The GPS observations in the Longmen Shan zone present unusual crustal uplifting, which is caused by the remote tectonic effects in the continent interior by the continent to continent collision of the Yangtze plate (Sichuan Basin) and the Tibet Plateau (Songpan-Ganzi Block). The dilatational and max-shear strain fields suggest that there exist double features as thrust and strike-slip fault in Longmen Shan region.

We present a model to show a potential motion of the materials that could be inferred from the upper crustal maximum shear patterns and tracing channel-flow throughout SET (Fig. 5). The resistance from the Sichuan Basin to the eastward expansion of SET resulted in crustal division, and meanwhile uplifted the Longmen Shan zone. Fortunately, the strike-slip faults and the passageway reconciled the whole crustal movements, from the upper rigid block to the lower crust. It presents a crustal subsidence that reveals lithospheric thinning and crustal extension along the southern Chuan-Dian block. Combining the GPS-derived 3-D velocity and strain patterns with lower crustal flow provides a potential tool to explain the crustal process of the block motion and the channel-flow features along SET.

## Methods

### GPS data and processing

**Figure 1:**
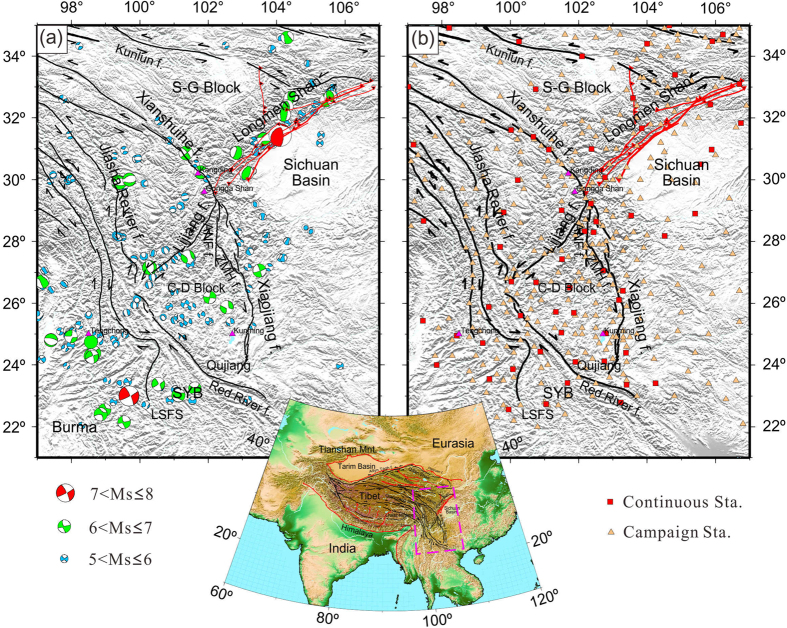
(**a**) Tectonic background in southeastern Tibet. The Earthquake focal mechanisms are from the Global CMT (Centroid Moment Tensor) catalogue from 1 January 1976 to 1 January 2016 in southwestern Tibet. (**b**) GPS sites location in the southeastern Tibetan Plateau relevant to this work, with records spanning from March 1999 to July 2016. The red squares denote the continuous GPS stations, and light yellow triangles denote the Campaign-mode stations, with records spanning from March 2009 to November 2015. Black lines are major active faults. ANH f.: Anninghe Fault; ZMH f.: Zemuhe Fault; S-G Block: Songpan-Ganzi Block; C-D Block: ChuanDian Block; SYB: Southern Yunnan sub-block; LSFS: Lanping-Simao Fold System. This figure is generated using the Generic Mapping Tools^26^.

**Figure 2:**
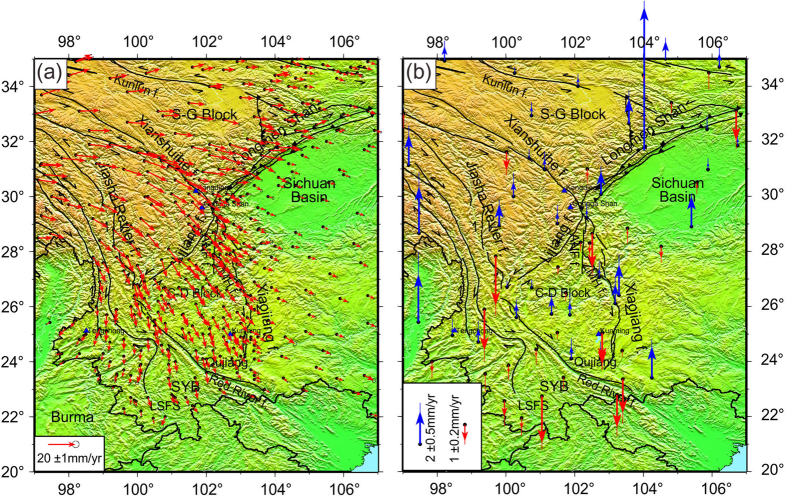
(**a**) GPS horizontal velocity field relative to stable Eurasia in SE Tibet. The light gray ellipse at the tip of each velocity vector is 95% confidence. The light gray error bar at the tip of each velocity vector represents 1 standard deviation. (**b**) The CGPS derived vertical velocity field relative to ITRF2008, the blue vectors denote uplift and red vectors denote subsidence. This figure is generated using the Generic Mapping Tools^26^.

We used 66 Continuous and 343 Campaign GPS stations (Fig. 1b) in and around SET from the Crustal Movement Observation Network of China (CMONOC I and II) to constrain the three-dimensional (3D) velocity field, which is presented in Fig. 2. Daily station positions are computed with the GIPSY6.2 software. We adopted a priori global pressure and temperature model (GPT)^31^ and the Global Mapping Function (GMF)^32^ for Troposphere correction. We considered the 1^st^ and 2^nd^ order effects in correcting the ionosphere effects. We applied International Earth Rotation and Reference Systems (IERS) 2010 conventions to correct the tidal solid Earth and pole tides^33^. The Finite Element Solution 2004 (FES2004) model with elastic Green’s Functions in the reference frame of the center of mass (CM) of the whole Earth system was used to correct for ocean tides and ocean tide loading^34^.

### Three-dimensional velocity field estimation

It allows re-estimation of sigma for GPS stations with two optional noise analysis models: white noise model and flicker noise plus white noise model^35^,^36^. To improve the vertical velocity from GPS time series, we used global mass loading models to correct the surface loads. We computed and removed the 3D Earth’s crustal displacements caused by mass loadings, including atmospheric loading, non-tidal ocean loading and hydrology loading models. We used the following models for various corrections: the MERRA model (Dataset: layer pressure thickness, air temperature, and specific humidity), OMCT model (Dataset: AOD1B) and MERRA model (Dataset: total water contents in soil at depths −2–0 m and water equivalent of snow mass) for atmospheric, non-tidal ocean loading and hydrology loadings^37^,^38^. The common mode errors (CME) were filtered by the Empirical Orthogonal Function (EOF) method from CGPS time series^39^,^40^. Finally, we used white noise model for campaign-mode GPS sites and flicker noise plus white noise model for continuous GPS time series. The data sets used in this study are provided in Supplementary Material (Tables S1 and S2).

### Strain Rates Calculation

**Figure 3:**
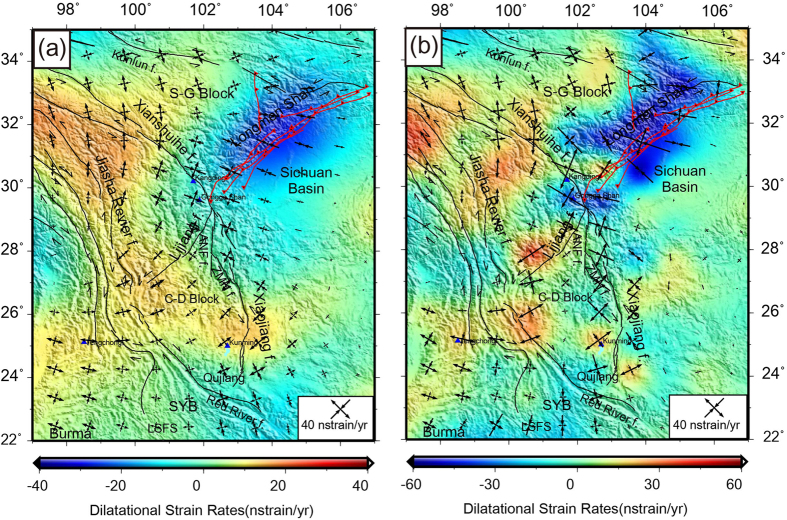
GPS derived principal strain rates (arrow pairs) and dilatational strain rate as continuous background color patterns using (**a**) the Grid-Distance Weighted routine with *α* = 100 km, and (**b**) the Grid-Nearest Neighbor routine with 12 neighbors and maximum radius = 300 km, respectively. Regional place names and locations are labeled by pink stars. Note that the colour scale bars are different in each sub-figure for the identification of regional strain patterns. This figure is generated using the Generic Mapping Tools^26^.

**Figure 4:**
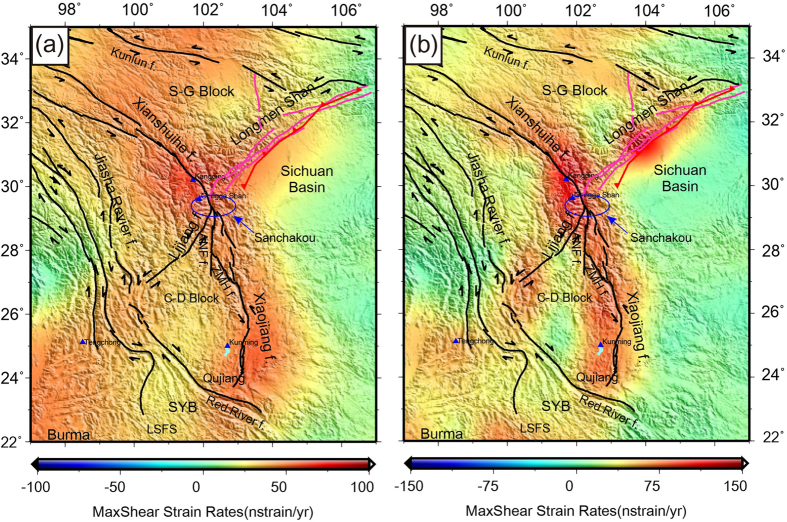
Max shear strain rates as background color patterns based on (**a**) the Grid-Distance Weighted routine with *α* = 100 km, and (**b**) the Grid-Nearest Neighbor routine with 12 neighbors and maximum radius = 300 km. Note the colour scale bars are different in each sub-figure for the identification of regional strain patterns. Blue ellipse denotes the location of Sanchakou. This figure is generated using the Generic Mapping Tools^26^.

**Figure 5:**
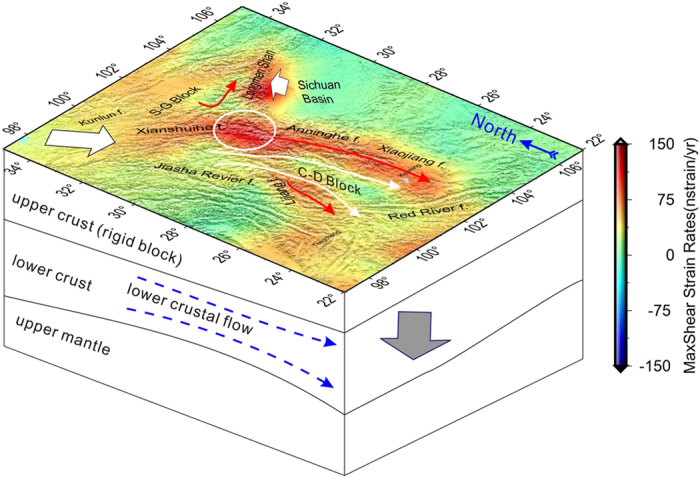
Sketch model of material migration, displaying the movements of the upper and lower crust in the South-Eastern Tibet (SET). The background shows the max-shear patterns. The red arrows denote the strike-slip faults, white arrows denote the directions of the upper rigid blocks, and the white ellipse denotes the location of Sanchakou. The blue arrow lines show the extending directions of the crustal flow, and gray arrow shows the crust subsiding in the southern C-D block. This figure is generated using the Generic Mapping Tools^26^.

We used the SSPX program (available at http://homepage.mac.com/nfcd/work/programs.html) to compute the strain field from GPS horizontal velocity data. More details including the main capabilities, functions and some example applications of SSPX were illustrated by Cardozo and Allmendinger^28^. Here, in order to conform the usage of this program in SET, we tested two optional strategies to compute the strain rates, namely using the grid-distance weighted and grid-nearest neighbor approaches. The nearest neighbor algorithm has a spatially variable distance weighting, which draws a regularly spaced grid and at the center of each square cell calculates the strain using the *n* nearest stations within a maximum radius, where *n* should be equal or greater than 3. The larger the number of nearest neighbors, the more the smoothing of the deformation field. In this study, we used Grid-Nearest Neighbor routine with 12 neighbors and maximum radius = 300 km for detailed strain modeling computation (Figs 3b and 4b). The larger scale strain patterns were computed by the Grid-Distance Weighted routine with 100 km grid spacing and *α* = 100 km. Weighting by distance (Grid-Distance) produces a smoother strain than the nearest neighbor method (Grid-Nearest Neighbor), and is particularly effective for visualizing regional patterns. However, it is likely that using the distance weighted approach some important details might be overlooked. The parameters setting for strain calculation are from Cardozo and Allmendinger^28^.

## Additional Information

**How to cite this article**: Pan, Y. and Shen, W.-B. Contemporary crustal movement of southeastern Tibet: Constraints from dense GPS measurements. *Sci. Rep.* **7**, 45348; doi: 10.1038/srep45348 (2017).

**Publisher's note:** Springer Nature remains neutral with regard to jurisdictional claims in published maps and institutional affiliations.

## Supplementary Material

Supplementary Information
