# Supplementary material for: Contemporary crustal movement of southeastern Tibet: Constraints from dense GPS measurements
Source: Sci Rep. 2017 Mar 28;7:45348. doi: 10.1038/srep45348 (PMC5368606; doi:10.1038/srep45348)
Supplement: Supplementary Information [file srep45348-s1.pdf]

Supplementary Information for
Contemporary crustal movement of southeastern Tibet: Constraints from dense GPS measurements

by Yuanjin Pan, Wen-Bin Shen

GPS three-dimensional velocity field data

Table S1 GPS derived horizontal velocities relative to Eurasia Fixed Reference Frame around the southeastern Tibet (velocity in mm/yr)

Station	Longitude	Latitude	V_N	σV_N	V_E	σV_E
F074_GPS	105.8	25	7.11	0.2	-2.91	0.32
F075_GPS	104.3	24.9	9.49	0.76	-6.58	0.84
F076_GPS	105.8	23.4	6.23	0.28	-1.9	0.15
F078_GPS	104.9	26.6	7.82	0.67	-4.32	0.48
F356_GPS	103.9	26.7	7.65	0.34	-4.4	0.22
F357_GPS	104.1	26.3	7.94	0.19	-3.42	0.23
F358_GPS	104.2	24.2	7.09	0.32	-2.78	0.18
F359_GPS	104.5	30.9	7.05	0.15	-2.26	0.31
F360_GPS	104.6	28.7	5.85	0.18	-3.16	0.32
F361_GPS	104.8	23	6.06	0.2	-2.1	0.16
F362_GPS	104.9	25.1	7.28	0.21	-3.68	0.36
F363_GPS	105	25.7	6.05	0.26	-2.61	0.42
F364_GPS	105	27.8	6.78	0.17	-2.17	0.19
F365_GPS	105.1	31.8	6.79	0.13	-1.33	0.13
F366_GPS	105.2	23.8	6.04	0.19	-2.63	0.3
F367_GPS	105.4	29.9	5.82	0.15	-2.05	0.16
F368_GPS	105.4	24.4	7.43	0.17	-3.18	0.28
F369_GPS	105.6	27.2	7.07	0.24	-3.63	0.29
F370_GPS	105.9	26.3	7.76	0.16	-3.48	0.23
F371_GPS	106.3	28.3	6.85	0.19	-3.62	0.23
F372_GPS	106.3	22.9	7.31	0.15	-2.75	0.26
F373_GPS	106.4	29.4	6.36	0.22	-2.02	0.18
F374_GPS	106.5	27.6	6.71	0.13	-3.95	0.27
F375_GPS	106.6	31.7	5.58	0.28	-2.31	0.3

F376_GPS	106.6	23.5	9.43	0.64	-4.88	0.76
F377_GPS	106.7	25.4	7.83	0.21	-3.27	0.27
F378_GPS	106.9	32.4	6.45	0.26	-1.58	0.34
G034_GPS	106.2	34.7	8.17	0.12	-2.07	0.23
G035_GPS	105.4	34.8	7.59	0.25	-1.88	0.16
G036_GPS	106.4	34.5	4.74	0.42	-4.42	0.28
G037_GPS	105.7	34.6	7.28	0.15	-2.67	0.16
G039_GPS	105.8	34.3	7.5	0.29	-2.65	0.3
G110_GPS	106.7	34.9	7.9	0.3	-2.65	0.3
G114_GPS	104.1	35	8.99	0.2	0.41	0.15
G116_GPS	104.5	35	7.81	0.28	0.7	0.25
G118_GPS	102.9	34.9	8.73	0.1	0.96	0.34
G119_GPS	104.5	34.9	8.55	0.43	-4.29	0.19
G120_GPS	104.9	34.7	7.95	0.2	-0.84	0.19
G121_GPS	104.9	34.5	8.13	0.15	-1.09	0.12
G362_GPS	106.6	34.7	7.76	0.34	-2.59	0.13
G364_GPS	101.9	34.6	11.02	0.18	2.37	0.17
G365_GPS	106.1	34.5	7.99	0.13	-2.15	0.19
H007_GPS	106.2	33.3	7.28	0.2	-2.33	0.29
H009_GPS	106	33	7.17	0.32	-1.76	0.29
H010_GPS	105.2	32.6	11.2	0.45	4.79	0.2
H011_GPS	105.8	32.4	5.95	0.25	-1.81	0.28
H012_GPS	105.5	32	6.14	0.22	-2.15	0.14
H013_GPS	103.2	34.7	10.63	0.15	0.1	0.33
H014_GPS	104.1	34.4	11.62	0.4	0.97	0.65
H015_GPS	102.5	34.6	10.98	0.17	0.46	0.37
H016_GPS	104.4	34	9.76	0.25	-1.54	0.22
H017_GPS	103.1	34.1	10.33	0.19	-0.58	0.25
H018_GPS	102.1	34	13.03	0.24	-0.94	0.28
H020_GPS	103.7	33.9	10.4	0.25	-0.85	0.26
H021_GPS	104.8	33.4	8.76	0.3	-1.57	0.39
H022_GPS	104.6	33	8.94	0.33	-1.13	0.28
H024_GPS	104.2	33.2	15.15	0.23	-4.04	0.12
H025_GPS	103.4	32.9	13.52	0.24	-3.11	0.21
H026_GPS	103	33.6	13.29	0.18	-0.91	0.15
H027_GPS	101.5	33.4	15.89	0.26	-1.22	0.29
H028_GPS	101.7	32.9	17.78	0.56	-2.5	0.69
H029_GPS	100.6	33.1	16.99	0.29	-0.02	0.42
H030_GPS	103.6	32.6	13.28	0.44	-4.95	0.2
H031_GPS	102.5	32.8	15.2	0.26	-4.35	0.3
H032_GPS	104.6	32.4	13.48	0.4	-1	0.24
H033_GPS	104.8	32.2	11.58	0.2	-2.24	0.26
H034_GPS	103.7	32.4	15.37	0.36	-3.71	0.13

H035_GPS	104.4	31.8	12.63	0.39	-3.07	0.21
H037_GPS	103.2	32.1	18.44	0.52	-5.05	0.31
H040_GPS	101.6	31.8	16.36	0.22	-3.7	0.41
H041_GPS	101.1	32.3	17.92	0.27	-2.29	0.46
H042_GPS	100.3	32.3	17.53	0.25	-1.71	0.27
H043_GPS	104.8	31.5	5.87	0.25	-2.96	0.18
H045_GPS	103.6	31.5	22.27	0.63	-4.62	0.21
H046_GPS	102.7	31.9	19.02	0.44	-6.45	0.3
H047_GPS	102.1	31.5	17.44	0.28	-3.07	0.2
H048_GPS	104.4	31.2	6.54	0.28	-1.2	0.19
H049_GPS	103.7	31.1	10.05	0.17	-5.93	0.3
H050_GPS	103.1	31	17.28	0.23	-10.95	0.31
H051_GPS	102.8	31	16.14	0.22	-5.2	0.49
H052_GPS	101.9	30.9	22.28	0.2	-2.75	0.2
H053_GPS	101.2	31	15.75	0.42	-5.85	0.2
H054_GPS	100.7	31.3	19.14	0.09	-6.77	0.14
H056_GPS	100.3	31.6	18.46	0.21	-4.74	0.38
H057_GPS	100.2	31.3	21.67	0.34	-7.19	0.43
H058_GPS	104.1	30.7	8.51	0.48	-5.09	0.78
H060_GPS	103.4	30.4	6.95	0.32	-5	0.61
H062_GPS	100.9	31.1	17.02	0.21	-7.03	0.37
H063_GPS	100.3	30.9	18.68	0.17	-9.01	0.39
H064_GPS	103.8	30	4.85	0.31	-1.49	0.57
H065_GPS	103	30	5.11	0.35	0.84	0.38
H066_GPS	101.8	30.1	12.66	0.28	-10.35	0.59
H067_GPS	101.5	30.1	13.9	0.2	-12.14	0.34
H068_GPS	101	30.1	15.59	0.29	-9.23	0.28
H069_GPS	99.2	30.1	16.32	0.25	-7.03	0.24
H071_GPS	104.6	30.4	6.33	0.08	-2.16	0.12
H072_GPS	102.8	29.8	7.17	0.23	-3.28	0.26
H073_GPS	102.3	29.8	8.34	0.14	-4.22	0.26
H074_GPS	101.6	29.8	12.18	0.32	-12.6	0.36
H075_GPS	100.4	29.7	15.1	0.14	-12.03	0.17
H076_GPS	103.5	29.6	7.23	0.34	-2.21	0.27
H078_GPS	102.1	29.7	11	0.17	-6.44	0.25
H080_GPS	100.1	29.2	13.66	0.11	-12.15	0.14
H081_GPS	103.3	29.2	6.72	0.24	-2.05	0.17
H082_GPS	102.4	29.3	10.56	0.25	-7.01	0.13
H083_GPS	101.5	29	11.12	0.19	-11.98	0.24
H086_GPS	103	28.8	6.9	0.22	-3.25	0.16
H087_GPS	102.8	29	8.22	0.32	-4.53	0.28
H089_GPS	103.1	28.3	8.5	0.21	-4.55	0.4
H090_GPS	102.5	28.7	9.01	0.29	-7.41	0.27

H091_GPS	102.4	28.3	9.77	0.41	-8.29	0.16
H092_GPS	102.1	28.5	10.38	0.19	-10.72	0.15
H093_GPS	103.6	28.3	9.77	0.66	1.39	0.81
H094_GPS	102.8	28	10.02	0.16	-6.52	0.15
H095_GPS	102.2	27.9	11.14	0.3	-10.89	0.26
H096_GPS	101.2	27.7	13.31	0.31	-10.83	0.23
H097_GPS	100.7	27.7	4.81	0.31	-16.72	0.3
H098_GPS	103.9	27.8	6.85	0.18	-3.73	0.22
H099_GPS	103.3	27.7	6.59	0.23	-3.67	0.24
H100_GPS	102.8	27.7	9.86	0.28	-7.09	0.29
H101_GPS	103.7	27.4	8.8	0.2	-2.54	0.12
H102_GPS	102.5	27.4	10.98	0.25	-10.24	0.22
H103_GPS	102.2	27.5	10.43	0.32	-11.97	0.5
H104_GPS	101.7	27.5	10.13	0.2	-10.96	0.51
H107_GPS	100.1	27.1	4.8	0.54	-14.89	0.3
H108_GPS	102.9	26.9	7.81	0.15	-8.58	0.19
H109_GPS	102.6	26.6	8.5	0.16	-11.93	0.13
H110_GPS	102.3	26.7	8.2	0.12	-11.53	0.15
H111_GPS	102.1	26.8	7.97	0.16	-10.12	0.23
H112_GPS	102	27	9.47	0.19	-12.53	0.29
H113_GPS	101.9	26.7	7.46	0.28	-11.73	0.17
H114_GPS	101.2	26.7	7.14	0.18	-12.05	0.26
H116_GPS	101.7	26.5	8.72	0.2	-12.89	0.21
H117_GPS	100.8	26.7	10.68	0.44	-12.24	0.28
H119_GPS	103.2	26.4	6.6	0.22	-6.74	0.14
H120_GPS	103.2	26.1	6.96	0.31	-7.13	0.18
H121_GPS	102.5	26	8.12	0.24	-12	0.12
H122_GPS	101.7	26.1	4.64	0.58	-13.01	0.17
H123_GPS	100.6	26.2	6.77	0.28	-10.6	0.16
H125_GPS	103.8	25.5	6.97	0.15	-3.87	0.27
H126_GPS	103.2	25.6	6.87	0.21	-7.27	0.23
H127_GPS	102.9	25.8	8.41	0.17	-11.04	0.17
H128_GPS	102.5	25.6	5.81	0.26	-11.31	0.13
H129_GPS	101.9	25.6	7.63	0.42	-8.47	0.45
H130_GPS	101.3	25.7	5.39	0.17	-11.08	0.21
H131_GPS	100.6	25.8	3.93	0.21	-10.69	0.09
H132_GPS	103.7	25	7.43	0.23	-4.26	0.14
H134_GPS	102.5	25.2	8.59	0.7	-12.53	0.72
H135_GPS	102.1	25.2	3.58	0.21	-12.49	0.14
H137_GPS	101.3	25.2	4.71	0.51	-12.46	0.37
H138_GPS	100.5	25.5	6.92	0.43	-14.51	0.89
H140_GPS	100.5	25.3	3.14	0.65	-11.34	0.32
H141_GPS	100.5	25	4.39	0.61	-10.96	0.21

H143_GPS	103.3	24.8	6.4	0.19	-5.87	0.22
H144_GPS	102.9	24.7	5.98	0.25	-8.11	0.22
H147_GPS	100.3	24.8	0.61	0.37	-10.9	0.24
H148_GPS	100.1	24.4	-1.02	0.4	-8.88	0.25
H150_GPS	102.4	24.2	2.56	0.22	-8.98	0.42
H151_GPS	102	24.1	2.94	0.14	-9.28	0.31
H152_GPS	101.1	24	1.75	0.38	-10.15	0.33
H153_GPS	100.9	24.4	0.68	0.3	-9.41	0.35
H154_GPS	103.4	24.1	7.33	0.19	-2.42	0.26
H157_GPS	102	23.6	1.54	0.28	-10.91	0.29
H160_GPS	100.9	23.9	1.47	0.15	-9.92	0.35
H162_GPS	100.1	23.9	-1.66	0.27	-8.94	0.42
H163_GPS	100.1	23.6	-3.69	0.74	-10.08	0.59
H164_GPS	103.4	23.5	5.94	0.24	-3.81	0.23
H165_GPS	103.3	23.7	6.37	0.47	-4.37	0.36
H167_GPS	102.4	23	6.41	0.47	-5.21	0.3
H170_GPS	103.7	23	5.27	0.33	-3.04	0.23
H177_GPS	100.8	22	-3.35	0.82	-9.08	0.92
H178_GPS	100.1	22.2	0.51	0.38	-4.9	0.22
H182_GPS	100	31.6	20.62	0.18	-4.87	0.33
H186_GPS	98.6	29.7	16.23	0.16	-6.63	0.3
H187_GPS	98.7	29.2	14.61	0.26	-8.51	0.17
H188_GPS	99.3	28.8	13.21	0.5	-11.99	0.23
H189_GPS	99.7	29	13.65	0.22	-9.15	0.65
H190_GPS	99.8	28.3	10.35	0.23	-13.68	0.24
H191_GPS	98.9	28.4	11.7	0.19	-10.77	0.19
H192_GPS	99.7	27.8	5.78	0.3	-15.24	0.25
H193_GPS	99	27.6	6.43	0.27	-11.01	0.22
H194_GPS	98.7	27.8	7.22	0.27	-9.7	0.2
H195_GPS	99.6	27.3	5.99	0.33	-13.82	0.23
H197_GPS	98.9	26.9	3.24	0.21	-10.32	0.17
H198_GPS	99.9	26.4	2.92	0.28	-12.64	0.24
H200_GPS	98.9	26.5	1.77	0.19	-10.1	0.29
H201_GPS	100	26.1	3.26	0.41	-14.05	0.28
H202_GPS	99.4	25.9	-1.65	0.17	-14.33	0.38
H203_GPS	98.8	26	1.07	0.33	-9.26	0.2
H204_GPS	99.9	25.7	2.21	0.57	-11.56	0.46
H206_GPS	99.1	25.7	-0.75	0.37	-11.63	0.43
H207_GPS	99.1	25.1	-0.86	0.31	-9.98	0.34
H209_GPS	99.9	24.6	0.24	0.38	-9.01	0.34
H210_GPS	99.6	24.8	0.09	0.2	-10.02	0.25
H212_GPS	98.3	24.8	-2.82	0.29	-5.57	0.22
H214_GPS	99.6	24.2	0.28	0.18	-8.06	0.14

H215_GPS	99.2	24	-1.56	0.31	-6.52	0.21
H216_GPS	98.8	23.8	-1.82	0.35	-5.67	0.2
H217_GPS	98.3	24.3	-4.04	0.32	-5.03	0.2
H219_GPS	99.8	23.5	-0.98	0.34	-7.73	0.61
H220_GPS	99.4	23.5	-1.61	0.43	-6.53	0.26
H223_GPS	99.3	23.1	-1.17	0.43	-2.85	0.59
H224_GPS	99.8	22.7	0	0.18	-6.18	0.3
H227_GPS	99.6	22.3	1.17	0.18	-6.14	0.27
H301_GPS	104.2	34.7	8.92	0.43	1.32	0.57
H302_GPS	104.6	34.3	8.87	0.3	-1.48	0.2
H303_GPS	103.9	34.2	10.56	0.27	-0.1	0.41
H304_GPS	104.9	34	7.87	0.12	-0.95	0.17
H305_GPS	105.1	33.5	7.71	0.24	-1.56	0.23
H307_GPS	97.8	24.3	-5.74	0.41	-1.17	0.78
H308_GPS	97.9	25.2	-6.51	0.43	-6.96	0.5
H309_GPS	98.2	25.5	-5.2	0.37	-6.88	0.21
H311_GPS	98.8	31.2	21.1	0.5	-3.83	0.18
H312_GPS	98.8	24.9	-2.45	0.3	-8.53	0.19
H313_GPS	98.9	25.2	-3.82	0.47	-10.17	0.25
H314_GPS	98.9	27.3	5.94	0.41	-10.51	0.29
H315_GPS	98.9	23.5	-2.12	0.33	-5.77	0.26
H316_GPS	99	29.7	14.72	0.3	-8.24	0.18
H317_GPS	99	24.3	-1.57	0.23	-6.88	0.23
H318_GPS	99.3	28.2	12.61	0.36	-11.79	0.32
H319_GPS	99.4	27.8	8.53	0.25	-12.04	0.16
H320_GPS	99.6	23.7	-1.44	0.35	-7.25	0.22
H321_GPS	99.6	31	19.09	0.45	-6.16	0.2
H323_GPS	99.8	26	1.62	0.23	-12.52	0.29
H324_GPS	99.9	26.8	3.39	0.16	-13.59	0.14
H325_GPS	100	26.8	4.02	0.31	-14	0.16
H327_GPS	100	27.5	6.72	0.5	-14.22	0.13
H328_GPS	100.2	26.3	6.04	0.61	-11.67	0.21
H329_GPS	100.3	30.5	16.39	0.37	-9.86	0.41
H330_GPS	100.3	28.6	11.26	0.2	-12.94	0.23
H331_GPS	100.3	22.7	2.94	0.29	-9.44	0.29
H333_GPS	100.5	27	6.01	0.18	-12.16	0.16
H334_GPS	100.7	26.4	5.39	0.21	-11.89	0.23
H335_GPS	100.7	24.6	1.41	0.32	-8.55	0.45
H336_GPS	100.8	25.4	1.46	0.32	-10.64	0.35
H337_GPS	100.9	28.1	10.44	0.17	-13.82	0.32
H338_GPS	101.1	29.8	10.54	0.6	-13.32	0.5
H339_GPS	101	22.8	1.25	0.39	-7.72	0.13
H340_GPS	100.9	26.1	5.44	0.23	-10.8	0.21

H341_GPS	100.9	23.4	2.04	0.21	-10.29	0.24
H342_GPS	101	27.6	9	0.15	-14.8	0.2
H343_GPS	101.1	30.6	18.12	0.2	-10	0.32
H344_GPS	101.4	22.7	3.04	0.3	-7.39	0.41
H345_GPS	101.4	30.6	18.12	0.18	-8.91	0.2
H346_GPS	101.4	27	9.4	0.2	-11.76	0.18
H347_GPS	101.5	29.3	11.68	0.19	-13.21	0.2
H349_GPS	101.5	27.7	11.19	0.23	-11.92	0.31
H350_GPS	101.6	22.4	5.13	0.25	-6.75	0.29
H351_GPS	101.6	25.3	6.64	0.12	-11.44	0.28
H352_GPS	101.7	24.4	1.79	0.15	-11.33	0.28
H353_GPS	102.2	24.4	2.33	0.17	-11.22	0.18
H354_GPS	101.7	23	1.58	0.31	-9.57	0.18
H355_GPS	101.8	30.6	10.61	0.18	-1.44	0.34
H356_GPS	101.9	25	2.71	0.22	-12.88	0.2
H357_GPS	101.9	28.2	9.86	0.27	-12.1	0.39
H358_GPS	102	28.6	11.82	0.17	-10.74	0.18
H359_GPS	102.2	27.8	10.75	0.12	-11.34	0.15
H360_GPS	102.1	25	4.64	0.47	-13.02	0.49
H361_GPS	102.2	30.1	9.58	0.26	-3.12	0.18
H362_GPS	102.2	25.6	6.12	0.17	-12.12	0.17
H363_GPS	102.2	29.4	10.44	0.16	-6.43	0.33
H364_GPS	102.2	23.9	2.53	0.22	-8.76	0.46
H365_GPS	102.2	28.7	10.31	0.25	-8.31	0.25
H366_GPS	102.3	29.1	9.11	0.21	-7.67	0.19
H367_GPS	102.4	27.9	9.85	0.31	-8.78	0.19
H368_GPS	102.4	29.9	8.34	0.36	-5	0.15
H369_GPS	102.4	27.6	10.06	0.19	-9.33	0.25
H370_GPS	102.5	23.4	5.24	0.32	-6.03	0.34
H371_GPS	102.5	24.9	3.91	0.23	-11.25	0.12
H372_GPS	102.6	30.1	9.63	0.3	-5.04	0.24
H373_GPS	102.6	27.9	10.24	0.32	-8.23	0.34
H374_GPS	102.6	24.7	3.59	0.15	-11.25	0.2
H375_GPS	102.6	28.6	9.79	0.12	-6.08	0.18
H376_GPS	102.7	27.9	9.82	0.26	-7.55	0.27
H377_GPS	102.8	30	7.01	0.16	-5.53	0.62
H378_GPS	102.7	28.5	9.83	0.22	-6.24	0.25
H379_GPS	102.8	25	4.76	0.27	-9.99	0.17
H380_GPS	102.8	24.5	3.98	0.2	-9	0.17
H381_GPS	102.8	23.9	4.46	0.16	-5.29	0.57
H382_GPS	102.9	23.6	3.32	0.16	-5.79	0.45
H384_GPS	102.9	30.5	11.42	0.55	0.51	0.31
H385_GPS	103	24.2	4.68	0.68	-6.11	0.24

H386_GPS	103	28.1	8.08	0.15	-5.93	0.25
H387_GPS	103	26.6	7.38	0.12	-8	0.21
H388_GPS	103	25.4	7.02	0.18	-8.45	0.27
H389_GPS	103	24.9	6.1	0.35	-7.31	0.14
H390_GPS	103.1	24.9	5.67	0.38	-5.87	0.28
H391_GPS	103.2	30.1	1.92	0.73	0	0.27
H392_GPS	103.3	25.1	8.65	0.14	-3.75	0.5
H393_GPS	103.3	24.9	5.78	0.27	-5.05	0.2
H394_GPS	103.4	28.1	5.74	0.25	-4.1	0.21
H396_GPS	103.4	26.9	6.42	0.33	-6.25	0.4
H397_GPS	103.4	24.6	6.74	0.28	-4.24	0.19
H398_GPS	103.4	25.9	6.48	0.17	-4.81	0.17
H399_GPS	103.5	24.9	7.3	0.31	-3.99	0.23
H400_GPS	103.8	29.4	7	0.19	-2.46	0.34
H401_GPS	104	28.2	7.22	0.23	-3.06	0.32
H402_GPS	104.1	30.4	5.83	0.18	-1.99	0.19
J001_GPS	101.6	34.7	11.91	0.27	3.07	0.15
J005_GPS	98.2	34.9	16.9	0.16	4.66	0.11
J006_GPS	99.7	33.8	18.25	0.12	2.01	0.19
J009_GPS	99.2	31.9	22.58	0.21	-3.19	0.13
J010_GPS	97.2	31.2	20.96	0.36	-1.88	0.41
J012_GPS	98	29.7	14.24	0.43	-2.95	0.68
J394_GPS	97.1	28.5	14.22	0.23	-0.08	0.14
J395_GPS	97.2	29.3	15.19	0.2	-4.51	0.21
J396_GPS	97.2	33.7	20.78	0.1	7.12	0.48
J399_GPS	97.5	34	18.61	0.1	5.15	0.16
J400_GPS	98	34.4	20.41	0.1	6.05	0.2
J402_GPS	98.2	31.5	22.35	0.35	-2.2	0.28
J403_GPS	98.6	32.4	20.41	0.28	-0.26	0.25
J404_GPS	98.6	35	16.08	0.16	3.94	0.11
J406_GPS	99.2	34.3	17.35	0.21	3.15	0.15
J408_GPS	99.7	32.7	19.09	0.17	-1.28	0.36
J410_GPS	101.2	31.3	15.33	0.35	-3.73	0.2
J411_GPS	102.1	31.9	16.47	0.44	-4.51	0.35
J412_GPS	102.5	31.4	19	0.63	-5.78	0.49
J413_GPS	102.8	31.6	19.57	0.61	-7.47	0.3
J414_GPS	104.7	30	5.92	0.29	-1.9	0.16
J415_GPS	103.7	32	16.6	0.43	-2.66	0.15
J417_GPS	104.1	31.8	19.74	0.64	-1.16	0.24
J418_GPS	104.3	32.8	11.34	0.49	-1.58	0.3
J419_GPS	105.3	33.2	7.38	0.28	-1.3	0.18
J420_GPS	98.3	33.1	19.74	0.1	0.85	0.4
J421_GPS	98.6	31.9	22.33	0.32	-2.61	0.41

J423_GPS	97.5	30	17.76	0.3	-4.36	0.19
J424_GPS	97.1	30.6	19.73	0.25	-2.54	0.3
J425_GPS	97.3	30.8	22.42	0.87	-1.57	0.28
J426_GPS	97.8	31.4	23.07	0.32	-2.52	0.27
JB23_GPS	106.7	33.1	7.2	0.22	-2.7	0.14
JB24_GPS	106	30.8	5.87	0.23	-2.18	0.33
JB25_GPS	106.7	26.4	6.41	0.23	-2.82	0.24
JB26_GPS	106.8	22.1	6.92	0.18	-2.09	0.23
JB33_GPS	103.9	33.3	12.23	0.18	0.48	0.15
JB34_GPS	102.3	31.7	18.04	0.2	-4.31	0.47
JB35_GPS	101.5	30.5	17.53	0.17	-8.84	0.3
JB36_GPS	103.5	28.8	7.54	0.2	-3.14	0.23
JB37_GPS	101.5	27.4	9.77	0.2	-11.14	0.23
JB38_GPS	100.2	26.9	4.73	0.39	-14.48	0.24
JB39_GPS	101.1	22.7	2.76	0.2	-7.06	0.23
JB40_GPS	100.3	30	15.88	0.21	-9.53	0.25
JB41_GPS	98.5	25	-3.82	0.21	-6.62	0.31
JB42_GPS	99.9	22.6	0.17	0.26	-4.25	0.37
GSLX_GPS	104.6	34.9	7.46	0.09	-0.1	0.08
GSMA_GPS	102.1	34	13.16	0.08	1.96	0.11
GSMX_GPS	104	34.4	13.42	0.24	3.22	0.19
GSQS_GPS	106.2	34.7	5.13	0.19	-0.75	0.13
GSTS_GPS	105.9	34.4	6.88	0.19	-1.96	0.23
GSWD_GPS	104.8	33.4	7.34	0.15	-0.69	0.15
MMMZ_GPS	97.4	25.4	-3.58	0.26	-1.84	0.19
QHBM_GPS	100.7	32.9	16.57	0.07	0.8	0.07
QHMD_GPS	98.2	34.9	14.67	0.26	6.31	0.12
QHMQ_GPS	100.2	34.4	13.92	0.06	4.5	0.07
QHYS_GPS	97	33	19.47	0.13	4.22	0.1
SCBZ_GPS	106.7	31.8	6.37	0.1	-1.46	0.08
SCDF_GPS	101.1	30.9	15.17	0.16	-6.11	0.14
SCGY_GPS	105.8	32.4	6.44	0.2	-1.07	0.16
SCGZ_GPS	100	31.6	18.67	0.12	-3.65	0.07
SCJL_GPS	101.5	29	11.78	0.18	-11.28	0.13
SCJU_GPS	104.5	28.1	6.67	0.11	-0.87	0.1
SCLH_GPS	100.6	31.3	17.95	0.1	-5.33	0.08
SCLT_GPS	100.2	29.9	15.68	0.11	-9.43	0.08
SCMB_GPS	103.5	28.8	5.89	0.2	-1.68	0.14
SCMN_GPS	102.1	28.3	10.66	0.11	-10.11	0.1
SCMX_GPS	103.8	31.6	16.88	0.14	0.2	0.11
SCNC_GPS	105.8	30.9	6.66	0.11	-2.11	0.09
SCNN_GPS	102.7	27	9.18	0.31	-10.54	0.22
SCPZ_GPS	101.7	26.5	7.77	0.16	-11.5	0.08

SCSM_GPS	102.3	29.2	10.32	0.18	-5.38	0.12
SCSN_GPS	105.5	30.5	6.65	0.13	-1.55	0.06
SCSP_GPS	103.5	32.6	12.94	0.16	-3.85	0.2
SCTQ_GPS	102.7	30	5.65	0.2	-8.19	0.26
SCXC_GPS	99.8	28.9	13.15	0.12	-11.28	0.09
SCXD_GPS	102.4	28.3	10.49	0.11	-7.95	0.09
SCXJ_GPS	102.3	31	13.27	0.1	-0.52	0.11
SCYX_GPS	102.5	28.6	9.77	0.24	-6.76	0.11
SCYY_GPS	101.5	27.4	10.35	0.1	-11.73	0.11
SNMX_GPS	106.7	33.1	8.22	0.16	-2.35	0.19
XZCD_GPS	97.1	31.1	21.37	0.14	-0.07	0.11
XZCY_GPS	97.4	28.6	13.65	0.3	-4.54	0.19
YNCX_GPS	101.4	25	4.34	0.24	-10.32	0.11
YNDC_GPS	103.1	26.1	6.86	0.19	-5.72	0.1
YNGM_GPS	99.3	23.5	-2.21	0.84	-5.53	0.29
YNHZ_GPS	103.2	26.4	6.82	0.2	-4.74	0.17
YNJD_GPS	100.8	24.4	1.46	0.56	-10.02	0.24
YNJP_GPS	103.2	22.7	6.52	0.17	-2.18	0.18
YNLA_GPS	99.9	22.5	0.39	0.35	-4.77	0.35
YNLC_GPS	100	23.8	1.45	0.29	-7.44	0.16
YNLJ_GPS	100	26.6	4.27	0.13	-12.77	0.1
YNMJ_GPS	101.6	23.4	2.91	0.23	-8.58	0.22
YNMH_GPS	100.4	21.9	0.81	0.22	-5.2	0.24
YNML_GPS	103.3	24.3	7.28	0.24	-2.23	0.24
YNMZ_GPS	103.4	23.3	6.02	0.24	-2.43	0.18
YNRL_GPS	97.8	24	-4.39	0.17	-2.96	0.14
YNSD_GPS	99.1	24.7	-1.26	0.23	-8.08	0.17
YNSM_GPS	101	22.7	1.15	0.23	-7.34	0.3
YNTC_GPS	98.4	24.9	-3.2	0.36	-5.12	0.17
YNTH_GPS	102.7	24.1	4.94	0.17	-6.96	0.18
YNWS_GPS	104.2	23.4	3.92	0.26	-3.78	0.21
YNXP_GPS	101.9	24.1	2.36	0.33	-9.48	0.48
YNYA_GPS	101.3	25.7	5.15	0.2	-10.4	0.09
YNYL_GPS	99.3	25.8	-0.12	0.25	-10.25	0.18
YNYM_GPS	101.8	25.6	5.84	0.12	-10.72	0.11
YNYS_GPS	100.7	26.6	7.56	0.12	-11.19	0.09
YNZD_GPS	99.6	27.8	5.79	0.35	-14.91	0.14
KMIN_GPS	102.7	25	5.28	0.09	-10.7	0.04
KUNM_GPS	102.7	25	3.35	0.12	-12.36	0.11
LUZH_GPS	105.4	28.9	5.66	0.08	-2.68	0.04
XIAG_GPS	100.3	25.6	1.6	0.15	-11.73	0.11

Table S2 GPS derived vertical velocities relative to ITRF2008 around the southeastern Tibet (velocity in mm/yr)

Station	Longitude	Latitude	V_U	σV_U
GSLX_GPS	104.6	35.0	1.07	0.20
GSMA_GPS	102.1	34.0	0.51	0.23
GSMX_GPS	104.0	34.4	-0.21	0.29
GSQS_GPS	106.2	34.7	0.82	0.45
GSTS_GPS	105.9	34.5	-0.02	0.98
GSWD_GPS	104.8	33.4	-0.37	0.27
KMIN_GPS	102.8	25.0	-1.90	0.25
KUNM_GPS	102.8	25.0	-1.70	0.53
LUZH_GPS	105.4	28.9	1.85	0.15
MMMZ_GPS	97.5	25.4	2.89	1.37
QHBM_GPS	100.7	32.9	0.57	0.24
QHMD_GPS	98.2	34.9	0.90	0.31
QHMQ_GPS	100.2	34.5	0.14	0.23
QHYS_GPS	97.0	33.0	-0.39	0.45
SCBZ_GPS	106.7	31.8	0.45	0.30
SCDF_GPS	101.1	31.0	0.67	0.40
SCGY_GPS	105.9	32.4	0.31	0.48
SCGZ_GPS	100.0	31.6	-1.11	0.31
SCJL_GPS	101.5	29.0	0.67	0.47
SCJU_GPS	104.5	28.2	-0.73	0.26
SCLH_GPS	100.7	31.4	0.25	0.29
SCLT_GPS	100.2	30.0	0.89	0.54
SCMB_GPS	103.5	28.8	-0.42	0.47
SCMN_GPS	102.2	28.3	-0.29	0.27
SCMX_GPS	103.8	31.7	8.66	0.39
SCNC_GPS	105.9	31.0	0.42	0.26
SCNN_GPS	102.7	27.1	0.52	0.50
SCPZ_GPS	101.7	26.5	-0.18	0.29
SCSM_GPS	102.4	29.2	0.49	0.43
SCSN_GPS	105.6	30.5	-0.43	0.28
SCSP_GPS	103.6	32.6	1.50	0.50
SCTQ_GPS	102.8	30.1	1.38	0.33
SCXC_GPS	99.8	28.9	1.31	0.26
SCXD_GPS	102.4	28.3	-0.61	0.29
SCXJ_GPS	102.4	31.0	-0.55	0.32
SCYX_GPS	102.5	28.7	-2.10	0.41
SCYY_GPS	101.5	27.4	-0.25	0.17
SNMX_GPS	106.7	33.1	-1.81	0.48

XIAG_GPS	100.3	25.6	0.84	0.26
XZCD_GPS	97.2	31.1	1.83	0.57
XZCY_GPS	97.5	28.7	2.82	0.87
YNCX_GPS	101.5	25.0	-0.11	0.25
YNDC_GPS	103.2	26.1	1.18	0.52
YNGM_GPS	99.4	23.5	-0.04	1.02
YNHZ_GPS	103.3	26.4	1.88	0.60
YNJD_GPS	100.9	24.4	-0.44	0.50
YNJP_GPS	103.2	22.8	-1.98	0.54
YNLA_GPS	100.0	22.6	-0.95	0.60
YNLC_GPS	100.1	23.9	-0.49	0.45
YNLJ_GPS	100.0	26.7	0.11	0.27
YNMH_GPS	100.4	21.9	-0.67	0.42
YNMJ_GPS	101.7	23.4	-0.14	0.55
YNML_GPS	103.4	24.4	-0.08	0.31
YNMZ_GPS	103.4	23.4	-2.07	0.63
YNRL_GPS	97.8	24.0	-0.66	0.37
YNSD_GPS	99.2	24.7	0.82	0.48
YNSM_GPS	101.0	22.7	-2.84	0.44
YNTC_GPS	98.4	25.0	0.42	0.57
YNTH_GPS	102.8	24.1	0.62	0.41
YNWS_GPS	104.2	23.4	1.90	0.41
YNXP_GPS	101.9	24.1	0.75	0.48
YNYA_GPS	101.3	25.7	1.02	0.25
YNYL_GPS	99.4	25.9	-2.48	0.70
YNYM_GPS	101.9	25.7	0.76	0.52
YNYS_GPS	100.8	26.7	0.19	0.30
YNZD_GPS	99.7	27.8	-2.98	0.62
